# Vitamin D supplementation decreases *Aspergillus fumigatus* specific Th2 responses in CF patients with aspergillus sensitization: a phase one open-label study

**DOI:** 10.1186/s40733-015-0003-5

**Published:** 2015-06-04

**Authors:** Nikki Lynn Hue Nguyen, Joseph M. Pilewski, Juan C. Celedón, Sivanarayana Mandalapu, Megan L. Blanchard, Adrienne DeRicco, Elizabeth Hartigan, John F. Alcorn, Jay K. Kolls

**Affiliations:** 1 Division of Infectious Diseases, Children’s Hospital of Pittsburgh of UPMC, Pittsburgh, PA USA; 2 Richard King Mellon Foundation Institute for Pediatric Research, Children’s Hospital of Pittsburgh of UPMC, Pittsburgh, PA USA; 3 Division of Pulmonary, Allergy and Critical Care Medicine, University of Pittsburgh, Pittsburgh, PA USA; 4 Antonio J and Janet Palumbo Cystic Fibrosis Center, Children’s Hospital of Pittsburgh of UPMC, Rangos Research Building, 4401 Penn Avenue, Pittsburgh, PA 15224 USA; 5 Department of Pediatrics, Division of Pulmonary Medicine, Allergy, and Immunology, Children’s Hospital of Pittsburgh of UPMC, Rangos Research Building, 4401 Penn Avenue, Pittsburgh, PA 15224 USA

**Keywords:** Vitamin D, Cystic fibrosis, Allergic bronchopulmonary aspergillosis

## Abstract

**Background:**

Patients with cystic fibrosis (CF) complicated by allergic bronchopulmonary aspergillosis (ABPA) are vitamin D deficient and *in vitro* treatment with 1,25 (OH)_2_ vitamin D_3_ of CD4+ cells from CF patients with ABPA decreases *Aspergillus fumigatus*(Af)*-*induced Th2 responses. This Phase I clinical trial investigated the safety and effectiveness of daily vitamin D_3_ supplementation in CF patients with ABPA to reduce allergic responses and ABPA symptoms, and increase serum vitamin D levels.

**Methods:**

Seven patients ages 12 years and older with a clinical diagnosis of CF and ABPA with current evidence of Af sensitization received 4000 IU vitamin D_3_ (cholecalciferol) daily for 24 weeks. The primary outcome of the study was safety followed by the *Aspergillus* induced IL-13 response in CD4+ T cells to test the hypothesis that vitamin D supplementation is safe and reduces *Aspergillus* induced IL-13 responses in CD4+ T cells. Secondary outcomes included total IgE, *Aspergillus*- specific IgE, vitamin D levels, FEV_1_, urinary calcium/creatinine ratio, and cytokine production by *Aspergillus*-stimulated peripheral blood T cells.

**Results:**

Six months of vitamin D_3_ supplementation resulted in significant increases in serum 25-(OH) vitamin D level, and the treatment was well tolerated without evidence of vitamin D toxicity or hypercalcemia. There were no serious adverse events. Daily vitamin D supplementation led to significantly decreased *Aspergillus* induced IL-13 responses between the baseline visit and that at 24 weeks (p = 0.04). *Aspergillus*-specific IgE level was also significantly decreased after 8 (p = 0.035) and 24 weeks of daily vitamin D supplementation (p = 0.04).

**Conclusions:**

4000 IU vitamin D_3_ daily over a 24-week period is well tolerated in CF patients with a history ABPA and current evidence of Th2 immunity to Af. . Daily vitamin D supplementation was associated with reduced *Aspergillus* induced IL-13 responses from peripheral. . CD4+ T cells and *Aspergillus*-specific IgE levels, as well as increased serum vitamin D levels. This treatment was well tolerated and the study supports further investigation of the use of vitamin D supplementation in Th2 mediated diseases.

**Trial registration:**

This trial was registered at www.clinicaltrials.gov as NCT01222273.

## Background

Cystic fibrosis (CF) is the most common severely life-shortening genetic disease in people of mixed European descent, with lower prevalence in Hispanics, African Americans, and Asians. Due to impaired mucociliary clearance, CF patients are susceptible to fungal colonization in the lungs, as up to 50 % of patients culture fungus from respiratory secretions [1]. The most common fungal pathogen infecting CF patients is *Aspergillus fumigatus*. In one study, a striking 84 % of CF patients had *Aspergillus fumigatus-*specific IgG (Asp IgG) antibodies compared to 6 % in control subjects and 20 % in allergic asthmatics [2]. While an extremely high percentage of CF patients have exposure to *A. fumigatus*, this may or may not result in clinical disease. Impaired clearance of *Aspergillus fumigatus* in the respiratory tract of immunocompromised individuals can lead to clinical manifestations that include invasive aspergillosis, mycetoma, fungal bronchitis, or allergic bronchopulmonary aspergillosis (ABPA) [3]. In contrast to patients with clinically irrelevant colonization and other types of fungal disease, patients with CF and ABPA have allergic immunological responses to *Aspergillus fumigatus* antigens, which results in increased Th2 responses and increased IgE levels [4, 5].

Vitamin D deficiency is prevalent in the CF population due to inadequate absorption, impaired metabolism, and limited sun exposure [6]. We have previously shown that patients in our CF center with CF and ABPA have significantly lower vitamin D levels than CF patients who do not have ABPA [7]. Further, *in vitro* treatment with 1,25 (OH)_2_ vitamin D_3_ decreased *Aspergillus* induced IL-13 responses from CD4+ T cells from ABPA patients [7]. In addition, it has been shown that a single, oral bolus of cholecalciferol (25,000 IU) increased serum-25 (OH) vitamin D concentrations and was associated with improved clinical outcomes in CF patients hospitalized with pulmonary exacerbations [8]. Further, a large, single dose of vitamin D (25,000 IU) in CF patients during pulmonary exacerbations was associated with a decrease in the inflammatory cytokines IL-6 and TNFα [9]. Taken together, these observations suggest that vitamin D plays a critical role in modulating immune responses. We therefore performed a Phase I, open label study to determine whether in CF patients with ABPA daily supplemental vitamin D is safe and reduces allergic response to *A. fumigatus*. Based on our prior work, we chose the *Aspergillus* specific IL-13 response as our primary immunologic outcome [7].

## Methods

### Study population

Subjects with CF with a prior history of clinically diagnosed ABPA were accrued from the Antonio J. and Janet Palumbo Cystic Fibrosis Center at the University of Pittsburgh Medical Center into the 24-week clinical trial of vitamin D supplementation (NCT01222273). To be enrolled into the study, subjects had to be 12 years and older, and have a diagnosis of CF based on standard criteria. In addition, subjects had to be clinically diagnosed with ABPA by a past or present respiratory culture for *A. fumigatus,* and have either a current IgE greater than 250 IU/ml or an *Aspergillus* specific-IgE (Asp IgE) classified as Class II or higher. Detailed inclusion and exclusion criteria are listed in Table 1. After meeting enrollment criteria at the screening visit, patients began the 24-week clinical trial of daily vitamin D supplementation with 4000 IU of vitamin D_3_ (cholecalciferol). During the trial, study subjects were seen 8 weeks (±7 days) and 24 weeks (±7 days) after beginning drug treatment.

**Table 1:** Inclusion and exclusion criteria for vitamin D supplementation trial

Inclusion criteria	Exclusion criteria
● ≥12 years male or female	● Systemic corticosteroids (1 mg/kg if <20 kg or >20 mg of prednisone per day)
● Confirmed CF diagnosis	
1. One or more clinical features consistent with CF phenotype AND (2 or 3)	● Investigational drug use within 30 days of screening
2. Positive sweat chloride >60 mEq/L	● Laboratory abnormalities at screening
3. Two identifiable mutations consistent with CF	● Serum calcium > 11 mg/dL
● Written informed consent (or assent)	● 25 (OH) D > 50 ng/mL at screening
● Clinically stable at enrollment as assessed by site investigator	● Creatinine ≥ 1.5 or estimated GFR < 60 by Cockcroft-Gault or MDRD equation
● Past or present respiratory culture positive for *Aspergillus fumigatus*	● LFT ≥ 3x ULN
● IgE ≥ 250 and/or presence of Class II or higher Aspergillus specific IgE on enrollment	● History or transplantation or currently on transplant list
● Ability to comply with medication use, study visits and study procedures as judged by site investigator	● Positive serum pregnancy test at screening
	● Pregnant, breastfeeding, or if post-menarche female, unwilling to practice birth control during participation in study
	● Presence of a condition or abnormality that would compromise safety of subject or quality of data
	● Diagnosis of HIV and a CD4+ T cell count < 500 cells/mL or active hepatitis C infection
	● Undergoing therapy for non-tuberculosis mycobacterial infection

On the basis of our prior findings [7], we designed this Phase I clinical trial to determine whether vitamin D supplementation is safe and reduces allergic responses to *Aspergillus* in CF patients with ABPA. Our primary outcome was thus safety, followed by the *Aspergillus-*induced IL-13 response in CD4+ T cells. Secondary outcomes included total IgE, *Aspergillus*-specific IgE, vitamin D level, FEV_1_, and cytokine production by *Aspergillus-*stimulated peripheral blood T cells. Clinical assessments at each time point are listed in Table 2.

**Table 2:** Timeline of clinical trial visits and assessments

	Timepoint					
Assessment	Screening	Visit 1	Visit 2	Phone call	Visit 3	Follow-up visit
	Day-7 to 28	Day 0	Day 55 ± 7	Day 112 ± 7	Day 182 ± 7	Day 196 ± 7
Informed consent	x					
Demographics/Medical history	x					
Concominant medications	x	x	x	x	x	x
Abbreviated physical exam	x				x	x
Vital signs	x	x			x	x
Spirametry	x				x	
Clinical laboratory assessments	x				x	
Blood collection for CF genotype	x					
Blood collection for HLA typing	x					
Blood for banking (immune monitoring)	x				x	
Adverse event assessments		x	x	x	x	x
Medication and diary drug		x				
Review study drug count and diary			x		x	

The Institutional Review Board at the University of Pittsburgh approved the trial, and patients provided written informed consent and were enrolled in the trial by one of the primary investigators.

### Vitamin D supplementation

After meeting enrollment criteria, patients were given the study drug. Vitamin D_3_ (cholecalciferol) was dispensed as 1000 IU tablets, and patients were instructed to take four tablets daily.

### Cell harvest

Human CD4+ and CD11c + cells were obtained from whole blood using Vacutainer CPT tubes (BD Pharmingen, Franklin Lakes, NJ) and re-suspended in cell buffer comprised of EDTA (Gibco, Grand Island, NY), BSA (Sigma, St. Louis, MO), and 1X PBS (Fisher, Waltham, MA). All cells were isolated by magnetic bead activated sorting using microbeads and MidiMacs (Miltenyi, Auburn, CA). The CD4+ cells were first isolated by positive separation in MS columns using CD4 Microbeads (Miltenyi). CD11c + cells were subsequently isolated from the negative fraction of the CD4+ isolation using Anti APC Microbeads then CD11c-APC microbeads for the final magnetic separation. DCs were then plated in flat bottom 96 well plates at a density of 5×10^5^ and 5×10^4^ DC cells per well in medium containing RPMI, L-glutamine, pen strep, FBS (Gibco), and Human AB serum (Atlanta Biologicals, Lawrenceville, GA). Cells in the plate were then stimulated with the following stimulators: TSLP (5 ng/mL, R and D Systems, Minneapolis, MN) and/or Aspergillus extract (ASPEXT, 1 μg/ml) (Holister-stier, Spokane, WA). One well in the plate was left un-stimulated as a control. 5×10^5^ CD4+ cells were then added. Control wells received CD4+ T-cells that were cultured in media or stimulated with CD3/CD28 beads (Dynal/Invitrogen, Grand Island, NY). Additionally, recombinant IL-2 was added to all wells on the plate (7.5 ng/ml). Medium containing RPMI, 5 % L-glutamine, 5 % pen strep, 10 % FBS (Gibco), and 5 % Human AB serum (Atlanta Biologicals) was then added to each well to reach final volume. All cells were then incubated at 37 °C and 5 % CO_2_ for 96 h. In some experiments, anti-human IL-10 (1 μg/ml final concentration) or recombinant human TGF-B sRII Fc Chimera (10 μg/ml final concentration) were added.

### Analytical assays

IL-4, IL-5, IL-10, IL-13, and IL-17 were measured by Luminex (Millipore, Billerica, MA) according to manufacturer’s instructions. The data was analyzed with Bio-Plex Manager software (Bio-Rad, Hercules, CA). To control for percentage of Aspergillus specific CD4+ T-cell in peripheral blood we normalized the APSEXT response to the CD3/CD8 stimulated response as this represents the maximal cytokine response in the culture system.

### Clinical laboratory assessment

Serum was collected and 25-OH vitamin D, IgE, Asp IgE, and calcium levels were determined by Sunquest Clinical Labs. Baseline total IgE and Asp IgE levels are reported from the screening visit. In addition, we measured Ca, Phosphorous, ALT, AST, total bilirubin, alkaline phosphatase, BUN, Cr, serum HCG, and IgG in all or a subset of the study participants. Urinary calcium and creatinine were measured at each time point and urinary calcium:creatinine ratio was calculated to assess for potential hypercalciuria (an early sign of vitamin D toxicity).

### Lung function

Lung function was measured using standard pulmonary function tests according to American Thoracic Society guidelines. Forced expiratory volume in 1 s (FEV_1_) was measured and the percent of predicted FEV_1_ (FEV1 % predicted) was calculated based on patient’s age and height using NHANES-3 reference values ^25^.

### Statistical analysis

All statistical analyses were performed using a one-tailed paired *T*-test or one-way ANOVA with Prism software (GraphPad).

## Results

Seven ABPA patients completed all time points in the study. The main characteristics of these subjects are shown in Table 3. There were nearly equal numbers of males and females, and study participants had the most common CF genotypes.

**Table 3:** Demographics of patients enrolled in vitamin D supplementation trial

Measurement	Value
BMI	22.5 ± 10.68 (17.25–30.75)
Age (years)	21.91 ± 2.69 (13.10–30.83)
Sex	4 female; 3 male
IgE (IU/mL)	344.6 ± 284.9 (142–835)
Asp IgE (kUA/I)	18.4 ± 14.7 (4.24–37.5)
Genotype	ΔF508/R553X; ΔF508/612+ 1G-T; neg/ΔF508; ΔF508/ΔF508; MEG/G542X; ΔF508/1213delT; ΔF508/R1162X

### Vitamin D supplementation in CF patients with ABPA is well tolerated and safe

**Fig. 1:**
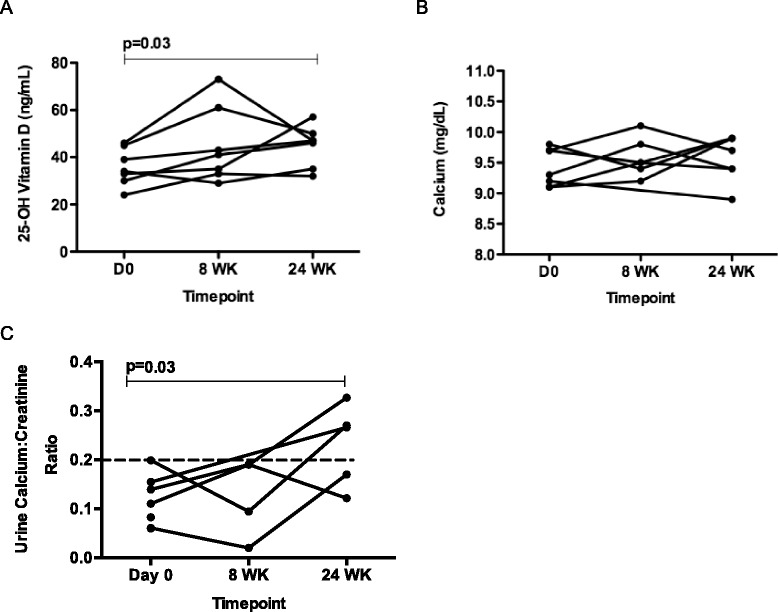
Vitamin D_3_ supplementation increases serum 25-OH vitamin D and does not impact serum calcium or lung function. **a**) Vitamin D levels increased over the 24 weeks of supplementation but were only significantly higher between day 0 and the 24-week time point. **b**) Serum calcium levels did not change significantly over the 24-week trial period. **c**) Urine calcium:creatinine ratios increased slightly over 24-weeks and were significantly higher between day 0 and 24-weeks. Data is graphed as for each individual patient at each time point P

At the initial screening visit, study participants had serum vitamin D (25-OH vitamin D) concentrations of 35.86 ± 3.02 ng/mL (Fig. 1). After 8 weeks of daily supplementation with 4000 IU of vitamin D, serum 25-OH vitamin D concentrations increased to 45.00 ± 6.09 ng/mL. At the end of the 24-week trial, serum 25-OH vitamin D concentrations were significantly increased to 44.86 ± 3.262 ng/mL (Fig. 1). A compliance table is listed as Table 4. Serum calcium levels did not change significantly over the 24-week trial period (Fig. 1), with average calcium levels of 9.41 ± 0.116 mg/dL at baseline and 9.59 ± 0.142 mg/dL at 24-weeks (Fig. 1). There was a statistically significant increase in urinary calcium/creatinine ratios with an increase in 3 of 7 patients over the 24-week trial period (Fig. 1) but these increments were not associated with any symptoms. There were also no serious adverse events reported, indicating this vitamin D supplementation is safe and well tolerated.

**Table 4:** Compliance by pill count

ID	Age	Started drug	Stopped drug	# days	Should have taken	# dispensed	# returned	# taken	Compliance by pill count (%)	Vit D dose at enrollment
1	25	10/21/2010	4/6/2011	168	672	750	110	640	95	400 QD
2	23	6/5/2011	12/9/2011	188	752	750	2	748	99	400 QD
3	30	10/2/2010	4/27/2011	208	832	750	0	?	?	400 QD
4	21	1/27/2012	7/25/2012	180	720	750	58	662	92	50,000 TIW
5	24	4/25/2012	10/3/2012	162	648	750	102	648	100	ABDEK 2/day plus vitamin D 1000 units and 400 units once daily
6	12	3/10/2011	8/17/2011	161	644	750	121	629	98	50,000 BIW
7	13	3/8/2011	9/12/2011	189	756	750	29	721	95	50,000 Qweek

### Effect of vitamin D supplementation on aspergillus-induced IL-13 responses

**Fig. 2:**
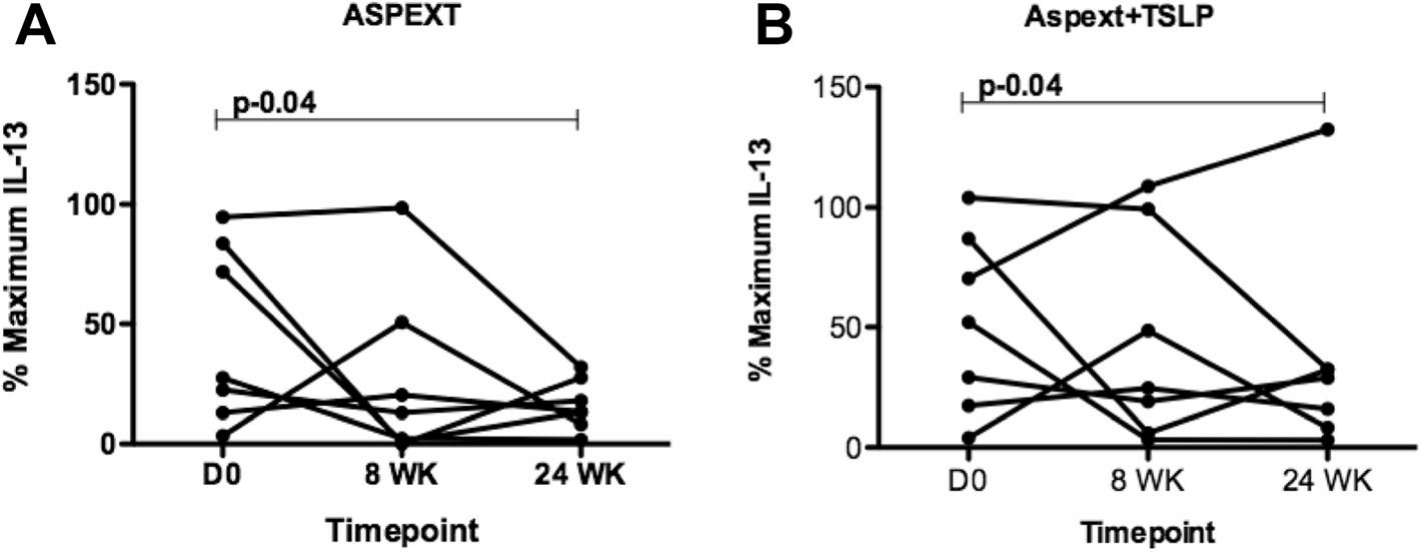
Vitamin D supplementation decreases Aspergillus induced IL-13 responses in CD4+ T cells. CD11c + DCs with from patients with confirmed ABPA before (D0) and 8 and 24 weeks after 4000 IU cholecalciferol daily were treated with **a**) media or **b**) TSLP (5 ng/ml) and then pulsed with Aspergillus extract. Purified CD4+ T-cells were added for 96 h. Supernatants were harvested and analyzed by Luminex for IL-13 production. Data is graphed as a percentage of the stimulated response (CD3/CD28) for each individual patient at each time point

We next determined whether vitamin D supplementation affected *Aspergillus* induced IL-13 responses in peripheral CD4+ T cells*.* At each time point in the vitamin D supplementation trial, peripheral blood was drawn and CD4+ T cells and CD11c + DCs were isolated by magnetic separation. CD11c + DCs were pulsed with *A. fumigatus* extract (ASPEXT), and CD4+ T cells were added and cultured for 96 h. In addition, some cells were cultured with TGFβ blocker or anti-IL10. To measure the maximal T-cell response, co-cultures were stimulated with human T-cell activating CD3/CD28 beads. Between the baseline visit and 8 weeks after daily cholecalciferol supplementation, there was a slight but statistically insignificant decrease in Th2 cytokine response to stimulation with ASPEXT measured by IL-13 cytokine response (Fig. 2). However, after 24 weeks on daily cholecalciferol, patients showed a significant decrease in IL-13 responses to ASPEXT (Fig. 2).

We have also previously shown that stimulating CD11c + DCs with TSLP increases Th2 response to *A. fumigatus* in CF patients with ABPA via upregulation of OX40L [10]. After 24 but not 8 weeks of vitamin D supplementation, TSLP-CD11c + DCs, co-cultured with autologous CD4+ T cells, also showed a significant reduction in the *A. fumigatus* IL-13 response (Fig. 2).

**Fig. 3:**
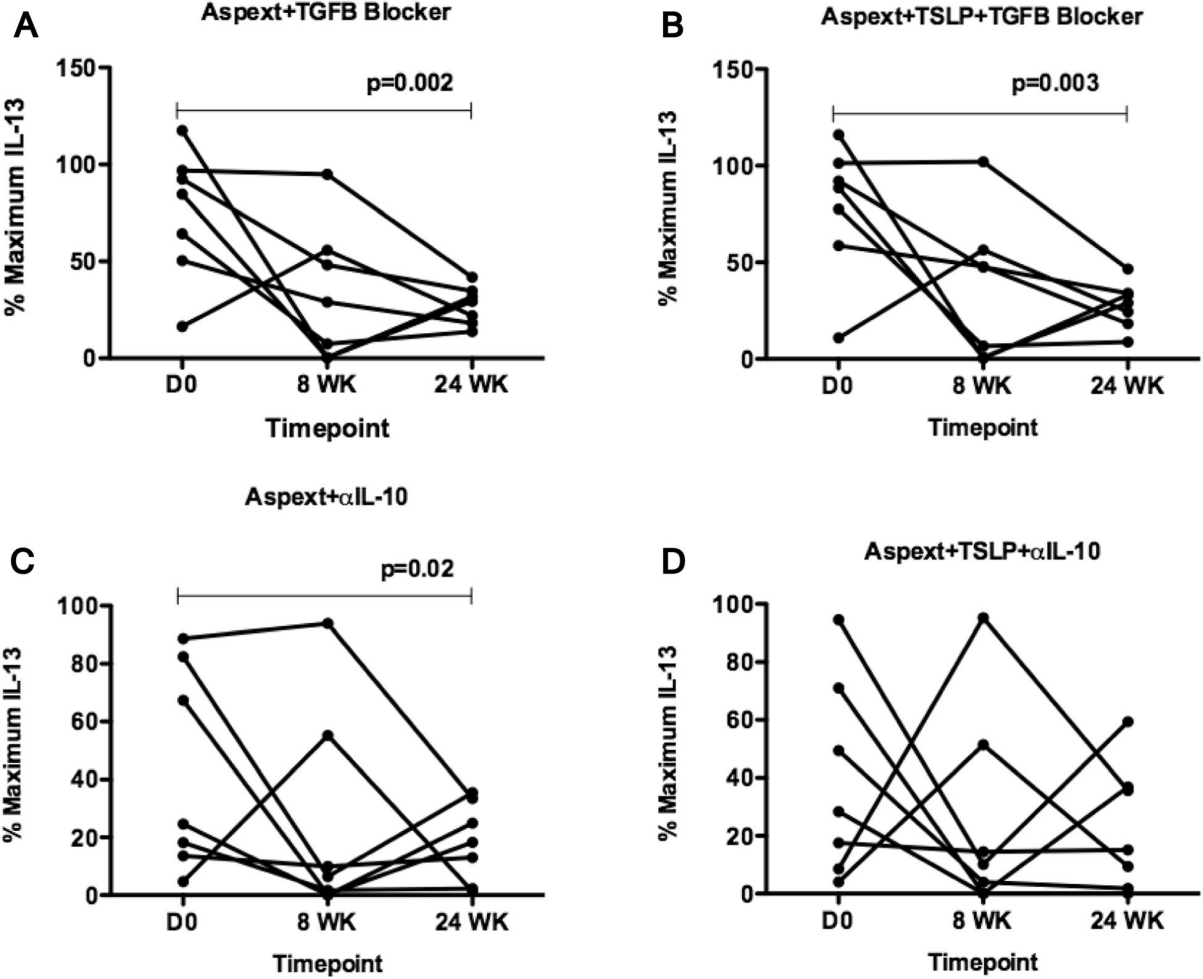
Blockade of TGFβ or IL-10 signaling does not reverse vitamin D mediated IL-13 cytokine production. CD11c + DCs with from patients with confirmed ABPA before (D0) and after supplementation with 4000 IU cholecalciferol daily were treated with **a** and **c**) media or **b** and **d**) TSLP (5 ng/ml) and then pulsed with Aspergillus Extract followed by addition of allogeneic bulk CD4+ T-cells followed by addition of **a** and **b**) sTGF-βII/FC (10 μg/mL) or **c** and **d**) anti-IL-10 (1 μg/mL). Cells were incubated for 96 h and IL-13 was measured in cell supernatants by Luminex. Data is graphed as a percentage of the stimulated response (CD3/CD28) for each individual patient at each time point

We have previously shown that *in vitro* 1,25 (OH)_2_ vitamin D_3_ treatment increases Treg cells and the blockade of TGFβ but not the Treg cytokine IL-10 *in vitro* attenuates the suppressive effects of 1,25 (OH)_2_ vitamin D_3_ on Th2 cytokine responses to *A. fumigatus* antigens [7]. We therefore determined whether blocking TGFβ or IL-10 would have similar affects in reversing vitamin D-mediated Th2 cytokine inhibition. To test this hypothesis, CD11c + DCs were stimulated with or without TSLP and pulsed with ASPEXT, followed by the addition of autologous CD4+ T cells. DC/T cell co-cultures were then incubated with sTGF-βII/FC or neutralizing antibody to IL-10 to antagonize TGFβ or IL-10 activity, respectively. Between baseline and 8 weeks, there was no significant change in *A. fumigatus* specific Th2 response, measured by IL-13, regardless of blockade of TGFβ or IL-10 (Fig. 3). However, contrary to our previous findings, after 24 weeks of vitamin D supplementation, *A. fumigatus* specific Th2 responses measured by IL-13 were still significantly decreased when TGFβ was neutralized (Fig. 3). This Th2 cytokine reduction after 24 weeks of vitamin D treatment occurred whether CD11c + DCs were pulsed with or without TSLP which is know to upregulate OX40L in CD11c + cells (Fig. 2). Neutralization of IL-10 did not affect CD4+ T-cell IL-13 responses from CD11c + DCs but the diminution in IL-13 responses (Fig. 3) in TSLP treated DCs failed to reach statistical significance suggesting that IL-10 may be playing a role in the observed reduced Th2 responses using these DCs (Fig. 3).

### Effect of vitamin D supplementation on secondary outcomes

**Fig. 4:**
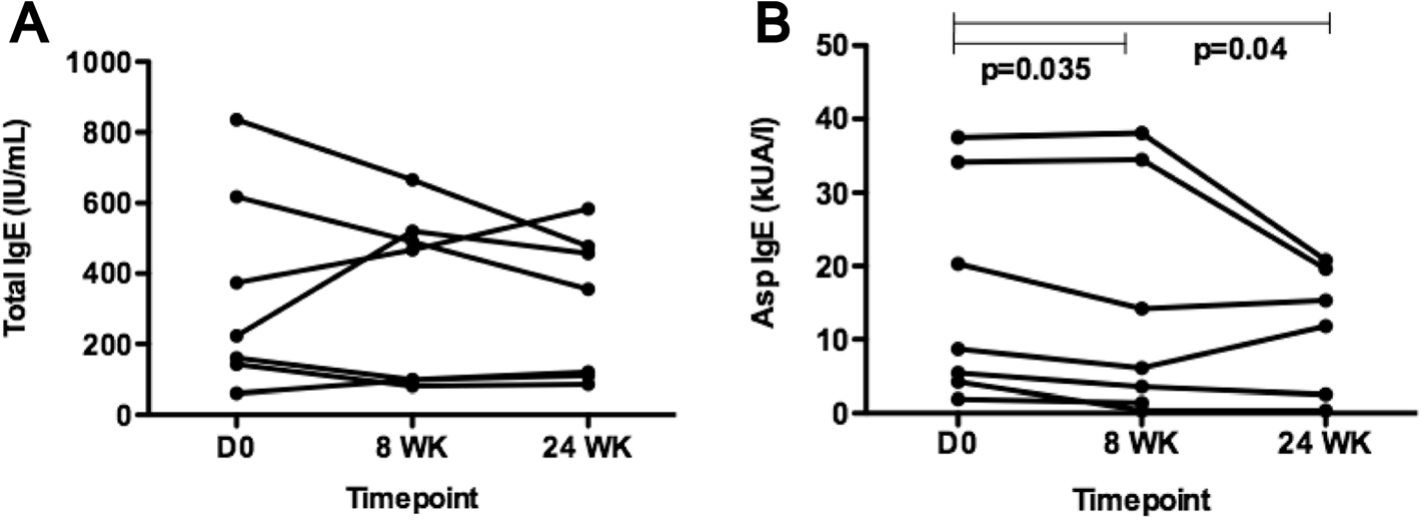
Effect of vitamin D on IgE and Aspergillus specific IgE. Daily vitamin D supplementation did not affect total IgE levels over a 24 week period (**a**) but vitamin D treatment significantly decreased Aspergillus specific IgE over 8 weeks and 24 weeks (**b**)

We have previously shown that our ABPA patients have high *Aspergillus* specific IgE and total IgE levels, which was associated with lower serum vitamin D levels [7]. In other allergic Th2-mediated diseases such as asthma, lower vitamin D levels have been shown to be associated with increased IgE levels and increased *Aspergillus* specific IgE levels [11, 12]. These data along with our own support the treatment of Th2-mediated diseases with vitamin D therapy. In our clinical trial, CF patients with ABPA had average total IgE levels of 344.6 ± 107.7 (standard error) IU/mL at the time of enrollment (Fig. 4). Total IgE levels decreased only slightly to 312.6 ± 77.66 IU/mL at the 24-week ending time point (Fig. 4). While total IgE levels did not significantly decrease, *Aspergillus*-specific IgE levels showed a significant reduction over the 24-week clinical trial period. At baseline, Asp IgE levels were 16.04 ± 5.594 kUA/I and significantly decreased to 14.03 ± 6.011 at 8 weeks after beginning daily cholecalciferol supplementation (Fig. 4). By the end of the 24-week clinical trial period, Asp IgE levels significantly decreased to 11.73 ± 3.581 (Fig. 4). Further, there were no significant changes in lung function measured by forced expiratory volume in 1 s in total FEV_1_ or FEV_1_ % predicted (data not shown).

**Fig. 5:**
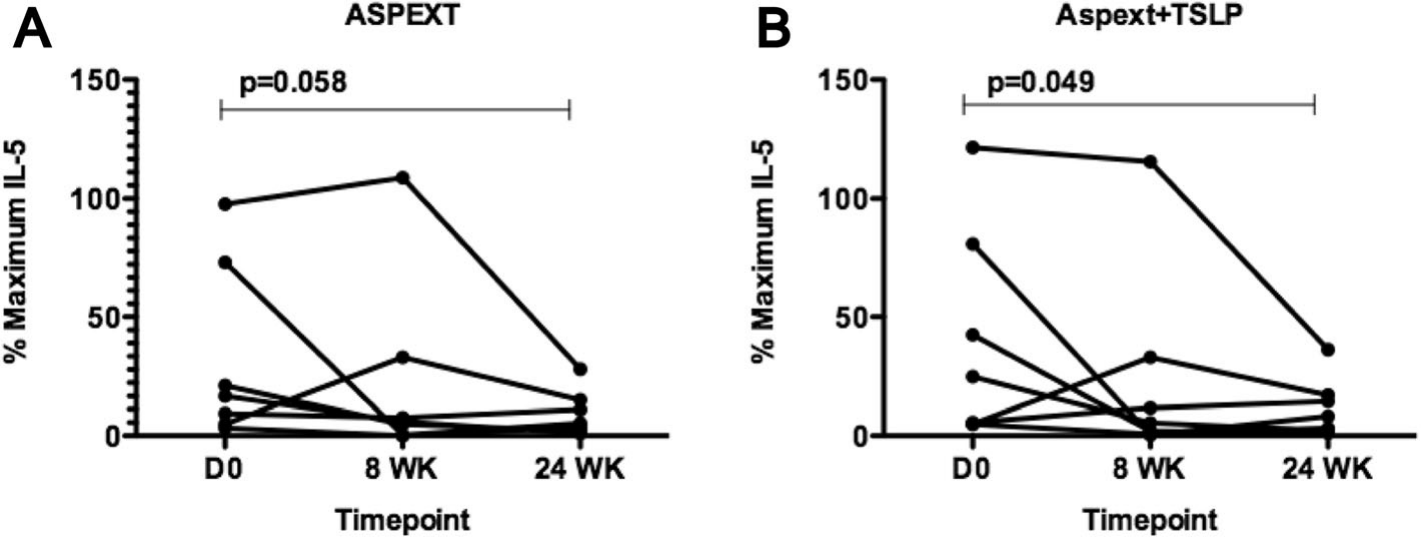
Vitamin D supplementation decreases *A. fumigatus* specific Th2 response. CD11c + DCs with from patients with confirmed ABPA before and 8 and 24 weeks after supplementation with 4000 IU cholecalciferol daily (n = 7) were treated with **a**) media or **b**) TSLP (5 ng/ml) and then pulsed with Aspergillus Extract. Purified CD4+ T-cells were added for 96 h. Supernatants were harvested and analyzed by Luminex for IL-5 production. Data is graphed as a percentage of the stimulated response (CD3/CD28) for each individual patient at each time point)

Finally, we also assessed *Aspergillus* stimulated IL-5 responses from peripheral CD4 + T cells. Between the baseline visit and 8 weeks after daily cholecalciferol supplementation, there was a statistically insignificant decrease in antigen stimulated IL-5 cytokine responses (Fig 5). However, after 24 weeks on daily cholecalciferol, patients showed a significant decrease in IL-5 responses to ASPEXT (Fig. 5). This reduction in IL-5 was also maintained when TSLP activated DCs were added to the culture (Fig. 5).

## Discussion

In most individuals, when benign antigens are inhaled, the lung responds with a tolerogenic immunological response [13–15]. However, in diseases such as ABPA, tolerance is lost or not established, and patients become sensitized to the inhaled fungal antigen *Aspergillus fumigatus.* We have previously shown that peripheral CD11c + DCs and TSLP-DCs from ABPA patients induce robust Th2 cytokine responses from autologous CD4+ T-cells in an OX40L-dependent manner [7]. In addition, in our CF cohort, vitamin D deficiency was associated with ABPA. *In vitro* treatment with 1,25-(OH)_2_ vitamin D_3_ reduced the robust Th2 response in patients with ABPA [7]. Based on data from our observational study, we initiated a clinical trial to assess the safety and the immunological effects of supplemental vitamin D_3_ (cholecalciferol) in patients with CF and ABPA.

This Phase I trial was implemented to test the safety and efficacy of vitamin D supplementation in CF patients with documented evidence of Af seinsitization. From a clinical perspective, the addition of daily vitamin D supplementation of 4000 IU significantly increased serum 25-OH vitamin D levels without vitamin D toxicity or hypercalcemia over the 24-week clinical trial period in CF patients with ABPA. It is important to note that this dose of vitamin D was prescribed on top of the patients regular vitamin D schedule (Table 4). Vitamin D dosing has moved beyond the typical 400 IU per day (Table 1) and this may explain why baseline vitamin D levels were higher in this cohort than our prior study [7]. As further evidence of compliance urine calcium:creatinine ratios increased to above 0.2 in 3 out of 7 patients at the 24-week time point. Typically, ratios above 0.2 may be indicative of hypercalciuria [16]. However, urine samples were collected at random during clinic visits. A 24-h urine collection would be more accurate at determining urine calcium excretion in our patients [16, 17]. After 24 weeks on daily vitamin D supplementation, patients had 25-OH vitamin D levels of 44.86 ± 8.630 ng/mL. These values are considered to be slightly above the minimal threshold (>30 ng/mL) of preferred 25-OH vitamin D concentrations recommended by the Cystic Fibrosis Foundation (CFF) [18].

The primary immunological outcome measure was the *A. fumigatus* specific IL-13 response in peripheral CD4+ T-cells. Over the 24-week period, IL-13 responses to *A. fumigatus* from DC/T-cell co-cultures significantly decreased. We have previously shown that *in vitro* 1,25-(OH)_2_ vitamin D_3_ increased CD4 + CD25 + TGFβ + Tregs [7] and others have shown that *in vitro* 1,25 dihydroxyvitamin D_3_ synergized to increase IL-10 in Tregs [19]. However, in this vitamin D supplementation study, blockade of TGFβ or IL-10 did not reverse inhibitory effects of vitamin D, suggesting that the decreased Th2 response in peripheral CD4+ T-cells after 24-weeks of vitamin D is not dependent on TGFβ or IL-10 in the *ex vivo* culture. We did not assess the frequency of FoxP3+ CD4+ T-cells in peripheral blood in this study and thus we cannot exclude an affect of vitamin D on the in vivo Treg population. It was recently shown that 1,25-(OH)_2_ vitamin D_3_ increased the expression the anti-microbial peptide LL-37 in CF epithelial cells along with decreasing inflammatory cytokines [20, 21]. LL-37 has been shown to have antimicrobial activity against microbes as well as fungi and viruses [22] and it has immunomodulatory effects on both epithelial cells and dendritic cell differentiation [23]. In addition to decreasing Th2 responses, one explanation for the decreased response to *A. fumigatus* with vitamin D supplementation may be due to an increase in antimicrobial peptides such as LL-37. While our clinical study had only 7 CF patients with ABPA who completed the 24-week vitamin D supplementation trial, we demonstrated that vitamin D supplementation reduced *A. fumigatus* specific IL-13 responses*.*

There are several limitations to the current study. First, prior to this vitamin D supplementation trial, the guidelines for 25-OH vitamin D concentrations were modified by the CFF. This may have contributed to the baseline Vitamin D levels being higher in this cohort prior to supplementation particularly in comparison to 25-OH vitamin D levels of 26.80 ± 15.32 ng/mL than in the initial observational study^7^, which were well below the current clinically recommended serum concentrations [18]. This may have limited our ability to detect an immunomodulatory effect of Vitamin D on *Aspergillus* responses. Despite the higher mean vitamin D levels in this cohort, *Aspergillus*-specific IgE levels decreased over the 24-week period. Second, the limited size of the study population did not permit us to track changes in other therapies for ABPA, such as prednisone and antifungal use that may have affected lung function and *in vitro* responses. Further studies are necessary to clarify this.

Others have shown that vitamin D deficiency is significantly and inversely associated with total IgE and Asp IgE [12, 24]. Additionally the presence of Aspergillus colonization itself has been shown to impair vitamin D receptor expression in CF [25]. Furthermore a recent clinical trial of vitamin D in asthma patients who were vitamin D deficient was negative in terms of vitamin D supplementation reducing time to first exacerbation or affecting FEV1 [26]. However Th2 immunity was not assessed as part of this trial. Moreover there was only measure of serum vitamin D levels which may not accurately measure VDR function which can be regulated by Aspergillus [25]. There is evidence that we impacted VDR function in this trial as measured by the increase in the urine calcium:creatinine ratio.

## Conclusions

Taken together with the results from this Phase I study, vitamin D supplementation may be beneficial in decreasing these allergic responses. These data provide rationale for increased vitamin D supplementation of CF patients with ABPA in addition to supporting further study of the effects of vitamin D supplementation to treat or prevent ABPA. Moreover fungal specific IL-13 responses may be a useful biomarker for future studies in patients with ABPA.

## References

[1] Pihet M, Carrere J, Cimon B, Chabasse D, Delhaes L, Symoens F (2008). Occurrence and relevance of filamentous fungi in respiratory secretions of patients with cystic fibrosis ‚Äì a review. Med Mycol.

[2] El-Dahr JM, Fink R, Selden R, Arruda LK, Platts-Mills TA, Heymann PW (1994). Development of immune responses to Aspergillus at an early age in children with cystic fibrosis. Am J Respir Crit Care Med.

[3] Romani L (2011). Immunity to fungal infections. Nat Rev Immunol.

[4] Moss RB (2005). Pathophysiology and immunology of allergic bronchopulmonary aspergillosis. Medical mycology: official publication of the International Society for Human and Animal Mycology.

[5] Agarwal R (2009). Allergic bronchopulmonary aspergillosis. Chest.

[6] Hall WB, Sparks AA, Aris RM (2010). Vitamin d deficiency in cystic fibrosis. Int J Endocrinol.

[7] Kreindler JL, Steele C, Nguyen N, Chan YR, Pilewski JM, Alcorn JF (2010). Vitamin D3 attenuates Th2 responses to Aspergillus fumigatus mounted by CD4+ T cells from cystic fibrosis patients with allergic bronchopulmonary aspergillosis. J Clin Investig.

[8] Grossmann RE, Zughaier SM, Kumari M, Seydafkan S, Lyles RH, Liu S (2012). Pilot study of vitamin D supplementation in adults with cystic fibrosis pulmonary exacerbation: A randomized, controlled trial. Dermato-endocrinology.

[9] Grossmann RE, Zughaier SM, Liu S, Lyles RH, Tangpricha V (2012). Impact of vitamin D supplementation on markers of inflammation in adults with cystic fibrosis hospitalized for a pulmonary exacerbation. Eur J Clin Nutr.

[10] Kulkarni R, Rampersaud R, Aguilar JL, Randis TM, Kreindler JL, Ratner AJ. Cigarette smoke inhibits airway epithelial cell innate immune responses to bacteria. Infect Immun. 2010;78:2146–2152. 10.1128/IAI.01410-09 PMC2863539 20194598

[11] Goleva E, Searing DA, Jackson LP, Richers BN, Leung DY (2012). Steroid requirements and immune associations with vitamin D are stronger in children than adults with asthma. J Allergy Clin Immunol.

[12] Gupta A, Sjoukes A, Richards D, Banya W, Hawrylowicz C, Bush A (2011). Relationship between serum vitamin D, disease severity, and airway remodeling in children with asthma. Am J Respir Crit Care Med.

[13] Ostroukhova M, Seguin-Devaux C, Oriss TB, Dixon-McCarthy B, Yang L, Ameredes BT (2004). Tolerance induced by inhaled antigen involves CD4+ T cells expressing membrane-bound TGF-Œ ≤ and FOXP3. J Clin Investig.

[14] Hammad H, Lambrecht BN (2008). Dendritic cells and epithelial cells: linking innate and adaptive immunity in asthma. Nat Rev Immunol.

[15] Montagnoli C, Fallarino F, Gaziano R, Bozza S, Bellocchio S, Zelante T (2006). Immunity and tolerance to Aspergillus involve functionally distinct regulatory T cells and tryptophan catabolism. Journal of Immunology (Baltimore, Md: 1950).

[16] Foley KF, Boccuzzi L (2010). Urine Calcium: Laboratory Measurement and Clinical Utility. Labmedicine.

[17] Jones AN, Blank RD, Lindstrom MJ, Penniston KL, Hansen KE (2010). Adjustment for body mass index and calcitrophic hormone levels improves the diagnostic accuracy of the spot urine calcium-to-creatinine ratio. Osteoporosis international: a journal established as result of cooperation between the European Foundation for Osteoporosis and the National Osteoporosis Foundation of the USA.

[18] Tangpricha V, Kelly A, Stephenson A, Maguiness K, Enders J, Robinson KA (2012). An update on the screening, diagnosis, management, and treatment of vitamin D deficiency in individuals with cystic fibrosis: evidence-based recommendations from the Cystic Fibrosis Foundation. J Clin Endocrinol Metab.

[19] Barrat FJ (2002). *In Vitro* Generation of Interleukin 10-producing Regulatory CD4+ T Cells Is Induced by Immunosuppressive Drugs and Inhibited by T Helper Type 1 (Th1)- and Th2-inducing Cytokines. J Exp Med.

[20] McNally JD, Leis K, Matheson LA, Karuananyake C, Sankaran K, Rosenberg AM (2009). Vitamin D deficiency in young children with severe acute lower respiratory infection. Pediatr Pulmonol.

[21] Yim S, Dhawan P, Ragunath C, Christakos S, Diamond G (2007). Induction of cathelicidin in normal and CF bronchial epithelial cells by 1,25-dihydroxyvitamin D(3). Journal of cystic fibrosis : official journal of the European Cystic Fibrosis Society.

[22] Liu PT, Stenger S, Li H, Wenzel L, Tan BH, Krutzik SR (2006). Toll-like receptor triggering of a vitamin D-mediated human antimicrobial response. Science (New York, NY).

[23] Bowdish DM, Davidson DJ, Scott MG, Hancock RE (2005). Immunomodulatory activities of small host defense peptides. Antimicrob Agents Chemother.

[24] Brehm JM, Celedon JC, Soto-Quiros ME, Avila L, Hunninghake GM, Forno E (2009). Serum Vitamin D Levels and Markers of Severity of Childhood Asthma in Costa Rica. Am J Respir Crit Care Med.

[25] Coughlan CA, Chotirmall SH, Renwick J, Hassan T, Low TB, Bergsson G (2012). The effect of Aspergillus fumigatus infection on vitamin D receptor expression in cystic fibrosis. Am J Respir Crit Care Med.

[26] Castro M, King TS, Kunselman SJ, Cabana MD, Denlinger L, Holguin F (2014). Effect of vitamin D3 on asthma treatment failures in adults with symptomatic asthma and lower vitamin D levels: the VIDA randomized clinical trial. Jounral of the American Medical Association.

